# Integrated Computational Pipeline for Single-Cell Genomic Profiling

**DOI:** 10.1200/CCI.19.00171

**Published:** 2020-05-20

**Authors:** Lubomir Chorbadjiev, Jude Kendall, Joan Alexander, Viacheslav Zhygulin, Junyan Song, Michael Wigler, Alexander Krasnitz

**Affiliations:** ^1^ Technological School of Electronic Systems, Technical University of Sofia, Sofia, Bulgaria; ^2^ Cold Spring Harbor Laboratory, Cold Spring Harbor, NY; ^3^ Department of Applied Mathematics and Statistics, Stony Brook University, Stony Brook, NY

## Abstract

**PURPOSE:**

Copy-number profiling of multiple individual cells from sparse sequencing may be used to reveal a detailed picture of genomic heterogeneity and clonal organization in a tissue biopsy specimen. We sought to provide a comprehensive computational pipeline for single-cell genomics, to facilitate adoption of this molecular technology for basic and translational research.

**MATERIALS AND METHODS:**

The pipeline comprises software tools programmed in Python and in R and depends on Bowtie, HISAT2, Matplotlib, and Qt. It is installed and used with Anaconda.

**RESULTS:**

Here we describe a complete pipeline for sparse single-cell genomic data, encompassing all steps of single-nucleus DNA copy-number profiling, from raw sequence processing to clonal structure analysis and visualization. For the latter, a specialized graphical user interface termed the single-cell genome viewer (SCGV) is provided. With applications to cancer diagnostics in mind, the SCGV allows for zooming and linkage to the University of California at Santa Cruz Genome Browser from each of the multiple integrated views of single-cell copy-number profiles. The latter can be organized by clonal substructure or by any of the associated metadata such as anatomic location and histologic characterization.

**CONCLUSION:**

The pipeline is available as open-source software for Linux and OS X. Its modular structure, extensive documentation, and ease of deployment using Anaconda facilitate its adoption by researchers and practitioners of single-cell genomics. With open-source availability and Massachusetts Institute of Technology licensing, it provides a basis for additional development by the cancer bioinformatics community.

## INTRODUCTION

Single-cell genomics is an indispensable tool in the study of genetically heterogeneous biologic systems such as cancer. Here we focus on one of the principal goals in the single-cell genomics field, namely, the detection of DNA copy-number variation (CNV) from sparse sequencing of single-cell nuclear genomes. A typical study conducted in this area would involve harvesting many hundreds of cells, often from multiple anatomic locations, each possibly affected by a tumor.^1,2^ Following a single-cell DNA preparation protocol and sequencing, CNV profiles are derived individually for each cell. A joint analysis of these profiles may then reveal the phylogenetic structure of the tumor cell population represented by the sample, with branches of the phylogenetic tree representing clonal subpopulations, each with a shared CNV pattern. Multiple computational steps are required to arrive at this phylogenetic description of the sample, starting from the raw sequence data. To facilitate this multistep processing, we here provide a description of a computational pipeline encompassing all the steps that are applied to single-cell genomic data. These data, presented as sequence reads from single cells, can be generated by a variety of molecular protocols. Visualizing the emerging single-cell population structure and placing it in a broader genomic and phenotypic context is often essential, in both research and clinical settings. For example, in prostate cancer, a diagnosis of localized disease may be reached on the basis of a histopathologic finding that cancer is confined to an index lesion detected by magnetic resonance imaging. Such a diagnosis would be challenged by single-cell genomic data if a clonal population of tumor cells, with massively altered genomes, were to be found to have spread away from the index lesion and into locations within the gland deemed cancer free by histopathology.^2^ The requisite functionality is provided by the single-cell genome viewer (SCGV), a graphical user front end for the pipeline. The SCGV integrates anatomic, histopathologic, and other cell-specific metadata with single-cell genomic profiles generated by the pipeline. Although elements of the pipeline were discussed in our earlier publications,^1-4^ here for the first time we present an integrated software framework for single-cell genomic profiling as a public resource incorporating these elements.

**CONTEXT**

**Key Objective**
We sought to facilitate the introduction of single-cell DNA copy-number analysis into clinical research and practice by providing a complete, easy to deploy and use, software pipeline for low-coverage single-cell genome sequence data processing and visualization.
**Knowledge Generated**
Analysis of single cells sampled from a tumor leads to the identification of copy-number events in the genomes of these cells. The tumor cell population structure is derived from collective examination of these altered genomes, together with quantitative measures of clonal complexity ubiquity and spread, and placed in a broader anatomic and histopathologic context by visualization.
**Relevance**
Genomic profiling of tumors at a single-cell level, facilitated by the current pipeline, is a novel source of information on the aggressive and invasive properties of tumors, with a potential for diagnostic usefulness when used in combination with findings from histopathology and imaging.

## RESULTS

**FIG 1.**
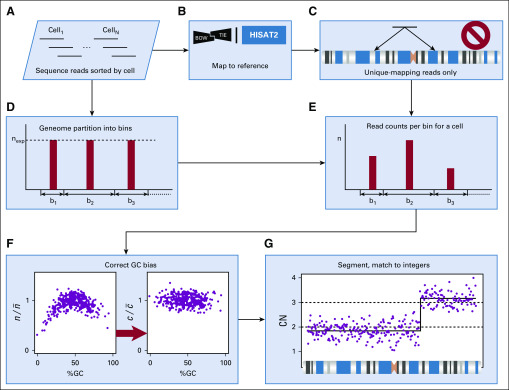
Single-cell–level data processing performed by the pipeline. Sequence reads sorted by cell (A) are mapped to the reference genome (B), and only uniquely mapping reads are retained for additional processing (C). Given the average read length determined from the read data (A), the genome is partitioned into contiguous bins with an equal expected number of uniquely mapping reads per bin (D). For each cell, the uniquely mapping sequence read counts per bin are computed (E). These counts exhibit systematic dependence on the guanine and cytosine (GC) content of the bin, which is removed using Lowess (F) to obtain a noisy estimate of *c*/$\bar{c}$ from *n*/$\bar{n}$ (see Derivation of the CN Profile of a Cell subsection). Segmentation is performed on the GC-adjusted read counts, and the result is rescaled to approximate integers (G).

**FIG 2.**
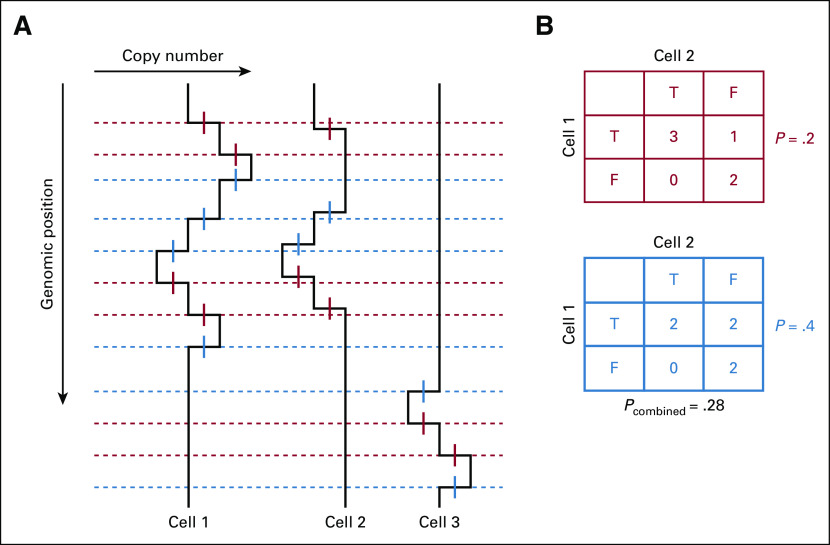
Computation of pairwise dissimilarities among single-cell copy-number (CN) profiles. (A) In a collection of 3 single-cell CN profiles, change points (CP) are identified and represented by short genomic intervals, shown in red for positive CP and in blue for negative CP. A set of genomic locations is found such that each CP interval contains at least one of them. Using these, a collection of CN profiles is reduced to 2 binary matrices, 1 for each CP sign, termed incidence tables. (B) From these, 2 contingency tables are formed for each pair of cells, and pairwise dissimilarity is computed by combining *P* values from the 2 Fisher’s exact tests.

**FIG 3.**
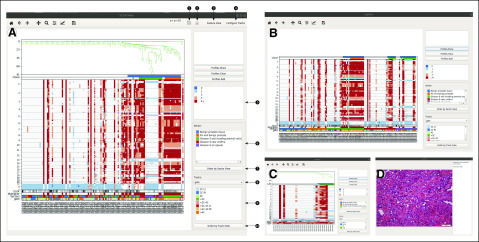
Functionalities of the single-cell genome viewer (SCGV), illustrated for a case of 130 cells harvested from multiple locations of a surgical specimen from a radical prostatectomy. (A) The opening view of the SCGV, with the genomic data and single-cell metadata loaded from a directory (1) or a compressed archive (2). Copy-number (CN) profiles of individual cells are shown as columns of a matrix, with genomic coordinates as rows. CN value is color coded (side panel 9). The user has the option (3) to also view the feature matrix, with features as defined by the change points, as explained in Results. The phylogenetic structure of the sample is visualized as a tree, shown in the upper portion of the view. The cells belonging to clones and subclones are indicated in color in the 2 tracks immediately under the tree. Additional tracks, in the lower portion of the view, display, for each cell, the mean squared deviation of a CN profile from a nearest integer, the value of a multiplier required to minimize that error, and the anatomic location (sector, color code shown in side panel 10) of origin for the cell. Additional tracks may be configured for the available single-cell metadata (4), such as the cell sorter gate for the cell and the color code for the tracks displayed (12, 13). (B) Cells may be reordered by sector (11) or by categoric values in any of the configurable tracks (14). (C) Any sector from side panel 10 may be selected to display it separately. (D) An annotated pathology slide from a sector may be displayed in a separate view.

**FIG 4.**
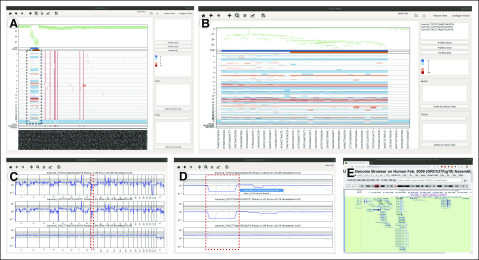
Additional functionalities of the single-cell genome viewer (SCGV), illustrated for a case of a sample of cells from the BJ euploid cell line with a 10% admixture of copy number (CN)–altered cells from the MKN-45 gastric cancer cell line. (A) The admixed MKN-45 cells form a clonal branch in the left portion of the main view. (B) This clone, and the corresponding portion of the heat map, may be examined in more detail using a zoom-in function. From this or any other view, the opening view may be restored by pressing the home button. Individual CN profiles may be selected (B, top side panel) and displayed in a stack (C). From this view, a genomic region of interest may be zoomed into (D) and displayed in University of California at Santa Cruz Genome Browser (E).

As illustrated in Figures 1 to 4, the pipeline accomplishes 3 major tasks: an estimation of integer-valued copy-number (CN) profiles of individual cells, starting from cell-specific genome sequencing read data (Fig 1); a collective analysis of multiple single-cell CN profiles to infer the clonal structure of the cell populations represented in the sample (Fig 2); and a graphical rendition of the output, complemented by nongenomic elements, such as histologic slide images of the tissues from which the cells originate (Figs 3 and 4). We next describe how each of these tasks is accomplished in turn.

### Derivation of the CN Profile of a Cell

The underlying assumption of CN profile estimation is that the CN at a given location in the genome is approximately proportionate to its read density. Our computational strategy, which is based on this assumption, is to make an equipartition (EP) of the genome into consecutive bins, such that the expected number of uniquely mapping sequence reads from a diploid cell would be the same for all bins (Fig 1A and 1D). EP facilitates signal aggregation across consecutive bins. Because the expected number of mapped reads per bin is dependent on read length, the binning scheme matches the average read length of the sample, and we determine EP by mapping large sets of in silico–derived reads.

There is however, another factor besides read length that alters the expectation of bin counts, namely, a bias caused by the guanine and cytosine (GC) content of the bin. This bias varies among single cell libraries. Thus, to maintain the principle of EP, we make an adjustment for the expected counts in a bin, separately for each cell. Adopting a method from CN analysis in the bulk,^5,6^ we use Lowess fit^7^ to compute the amount of adjustment, as explained in the following text.

The number of bins in the partition is user defined, and its optimal choice depends on the coverage depth. For diploid cells, we recommend aiming at binning schemes with an average read count of at least 20 per bin. We offer as an option masking centromeres from the partition. We recommend this because it is our experience that the uniqueness of mapping often cannot be determined accurately for the sequences in the centromeres.

The CN profile of a cell is determined in several steps. Sequence reads originating from the cell are mapped to the reference genome using HISAT2^8^ or Bowtie,^9^ and uniquely mapping reads increase the appropriate bin read count by an increment of one (Figs 1A, 1C, and 1E). We next perform GC bias correction, adopting for this purpose the following model:

$$\text{log }(n_{b}/\bar{n})=\text{log}(c_{b}/\bar{c})+B\left(g_{b}\right)+r,$$

where *n_b_* is the read count in bin *b* and *c_b_* is the CN of that bin. An overbar denotes the mean of the corresponding quantity over all bins. Furthermore, *B* is the bias term dependent on the bin GC content *g_b_*, and *r* is random noise with zero mean. We subtract a Lowess fit for *B*(*g*) from log(*n_b/_*$\bar{n}$; Fig 1F) and estimate log(*c_b_*/$\bar{c}$) from the residual log(*c_b_*/$\bar{c}$) + *r*. To do so, we approximate log(*c_b_*/$\bar{c}$) + *r* by a piecewise-constant function of the bin number *b*, a process known as segmentation (Fig 1G). For this purpose, we use, with minor modifications, the circular binary segmentation (CBS) algorithm, as implemented by the package DNAcopy.^10,11^ CBS and similar segmentation algorithms are probabilistic: positions of the segment boundaries, or CN change points (CP), are inferred to within a CI, centered at the most likely position of the CP.

The final step in CN derivation is taken in the expectation that the CN of a single cell is integer throughout the genome. To find the corresponding integer values, we find a multiplier *M* for the segmented CN ratio *c_b_*/$\bar{c}$, such that the mean squared error from rounding *M c_b_*/$\bar{c}$ to the nearest integer is minimized^12^ (Fig 1G). The corresponding nearest integer values are then taken as the final estimate of the CN *c_b_*, to be used in downstream analysis. In addition, the mean squared rounding error for the optimal *M* is a useful measure for quality control, because its anomalously large values may point to molecular protocol errors, such as DNA sequence reads from multiple cells being attributed to a single cell or single nuclear fragmentation or degradation. Finally, note that, with integer-valued *c_b_*, CP with equal flanking integer CN values are eliminated and the corresponding segments merged.

### Collective Analysis of CN Profiles and Derivation of the Clonal Structure

Integer-valued CN profiles of multiple cells are analyzed collectively to establish the clonal structure of the cell populations represented in the sample. To make this collective analysis possible, we compare single-cell CN profiles pairwise and quantify their dissimilarities. To do so, we use CP observed in CN profiles of the cells to define a set of features such that, for each feature and profile, the feature is either present in the CN profile or not. To define the feature set, we follow a procedure depicted in Fig 2. Namely, each CN profile is reduced to a series of CP (Fig 2A). A CP has 2 attributes: the genomic location or the number of the genomic bin where it occurs and the sign of the CN change because the CP is crossed in the direction of increasing genomic coordinate. Given the inherent uncertainty of the CP positions, they are represented by short genomic intervals (CP intervals), each centered at the most likely location of the CP, as inferred by segmentation. We next identify, for all such CP intervals in the entire collection of single-cell profiles corresponding to a given CP sign, the smallest set of points in the genome, called stabbing points, such that each CP interval contains at least one of these points. This is the simplest instance of a minimal stabbing problem,^13^ and it is solved by a greedy algorithm, with execution time of *O*(*k* log *k*), where n is the number of CP intervals. Each stabbing point in the resulting set defines a feature. A feature is said to be present in a cell genome if there is a CP of the appropriate sign in the CN profile whose CP interval contains the corresponding stabbing point. As a result, the collection of CN profiles is reduced to 2 binary matrices, 1 per CP sign. Each matrix has 1 column per cell and 1 row per feature. These matrices are called the incidence tables.

Represented in terms of features, the collection of cell genomes is amenable to phylogenetic analysis. We expect that 2 cells with a more recent common ancestor will share more features than will 2 cells with only a distantly related common ancestor. We therefore use feature sharing to quantify pairwise dissimilarity among cell genomes. We compute, for each pair of cells, two 2×2 contingency tables for the features, 1 for each sign, and, from each of these, the *P* value for Fisher’s exact test, with the alternative that the odds ratio is above 1. We then combine the two *P* values into one, *p_c_*, using either Fisher’s^14^ or Stouffer’s^15^ rule (Fig 2B), and define pairwise dissimilarity of the cell genomes as log(*p_c_*). With this dissimilarity, we compute the phylogenetic tree, with cells as leaves. We use hierarchic clustering, with user-defined linkage, to compute the tree.

As a final step in the inference of the structure of the cell population sampled, we identify highly cohesive branches of the phylogenetic tree, and we take these to represent clonal subpopulations. To find such branches, we first examine the statistical significance of dissimilarities in pairs of cell genomes. We do so by assuming the null distribution of dissimilarities to come from random incidence tables, whose marginals are the same as those observed, exactly for the rows and on average for the columns, separately for each CP sign. To compute the false discovery rate (FDR) for the observed dissimilarities, we sample from the null distribution by randomizing the incidence tables with the marginals constrained as described. We then set a threshold on FDR and declare a tree branch cohesive if FDR is below threshold for all pairwise dissimilarities of the cells on the branch. We say that a branch represents a “hard clone” if it (1) is cohesive, (2) does not have a cohesive parent, and (3) in addition, there is a certain minimal number of features that are present in no less than a preset percentage of cells on the branch. We further say that a branch represents a “soft” clone if it contains a hard-clonal branch among its descendants and satisfies condition 3, whereas its parent branch does not. To complete our analysis in the population structure, each of the detected clonal branches is examined for the presence of subclones. This is accomplished by applying the clone-finding procedure to the incidence tables for the cells in the clonal branch only, which is simplified by removing rows of all 1s or all 0s.

### Visualization

The SCGV is a visualization interface for a collection of single-cell genomes, together with the clonal structure it represents and additional relevant information, not necessarily genomic in origin. The key functionalities of the SCGV are illustrated in Figs 3 and 4. The data displayed in Fig 3 originate from a radical-prostatectomy specimen,^2^ which scored Gleason 9 on histopathologic evaluation. Cells for genomic analysis were harvested from a number of areas in the prostate. The metadata include the anatomic location of origin (sector) for each cell and a histopathologic evaluation for each location of origin. Genomic data in Fig 4 were generated using 10× Genomics single-cell CNV technology^16^ and represent a mixture of cells from BJ, a human diploid foreskin fibroblast cell line (ATCC CRL-2522) and MKN-45, a human gastric cancer cell line, with a 10:1 ratio.

Once the SCGV is invoked, the processed genomic data, together with histopathologic and other annotation, are read in, either from a directory or from a compressed file archive using the appropriate functions (Figs 3A and 4A). In the opening screen, single-cell genomes are displayed as columns of a heat map, with the chromosomes concatenated from 1 through Y. CN gains and losses are encoded in red and blue colors, respectively, with darker colors corresponding to greater deviations from CN 2. Alternatively, the heat map may be used to represent the incidence tables described in the preceding subsection, with positive and negative features present in each cell genome shown at their genomic position in red and blue colors, respectively (button 3 in Fig 3A, the resulting view not shown). The cells are arranged horizontally as leaves of a phylogenetic tree. The 2 tracks immediately above the heat map are used to indicate clonal and subclonal cell populations present in the data. Additional tracks are located below the heat map and are used to display user-supplied, cell-specific metadata. One of these is reserved to display the anatomic location of origin (sector) for each cell, if available. Other tracks are configurable by the user and can be used to display any categoric or numeric metadata associated with the cells. For example, a track may be used to indicate the cell type as determined by flow-cytometric analysis. The cells can be reordered by the values encoded in any of the tracks. A particularly useful reordering is by sector, making it easy to see the sectors in which clonal populations reside (Fig 3B). A subset of the data, and a corresponding subtree, may be displayed for any chosen value in any of the tracks, in particular, for any given sector (Fig 3C). An additional function of the SCGV facilitates the display of images associated with the sector, in particular, those of hematoxylin and eosin–stained tissue slides (Fig 3D). A user may select and zoom into subsets of cells, such as those representing a clone, and/or parts of their genomes, for closer examination (Fig 4B). Alternatively, it is possible to select any number of cells, either by location in the heat map or by typing their cell identifiers, and display a stack of their CN profiles (Fig 4C). These can also be zoomed into (Fig 4D), and the University of California at Santa Cruz genome browser can be invoked for a genomic region of interest (Fig 4E).

### Software Design and Organization

The complete pipeline is termed Sparse Genomic Analysis of Individual Nuclei by Sequencing (SGAINS) and contains modules in the Python and R languages, in addition to third-party tools. The R language modules of SGAINS encompass the downstream part of SGAINS, starting with segmentation of CN profiles, and are organized into an R package termed SCclust, available for download separately. Our visualization tool, the SCGV, is downloaded and installed as a separate unit.

In designing our tools, we emphasized flexibility of use and modularity. The user has the option to execute the entire pipeline, starting from unmapped sequence read data sorted by cell and minimally annotated reference genome sequence. In this case, the user can control the execution by means of approximately 50 parameters defined in the configuration file. Multiple additional parameters, either provided as command-line options for subcommands or as arguments of the functions composing the SCclust package, are then set to their default values. Alternatively, if a finer level of control is required, the pipeline may be executed interactively as a series of subcommands and SCclust function calls, with all parameters available to the user. Modularity also means that multiple subcommands, SCclust and the functions within it, and the SCGV can be used in a stand-alone fashion. For example, a user may use tools other than SCclust to discover the clonal structure in a set of single-cell CN profiles, then use the SCGV to visualize the result, with no loss of SCGV functionality. The SCGV, in addition, imposes only minimal data requirements on the user: multiple data elements, including the phylogenetic tree, the sector assignment, and the microscopic slide images may be missing without affecting the SCGV functionalities that are independent of these elements.

A small number of operations in the pipeline claim most of the execution time. We use parallelization to speed up these critical modules. At present, processing by multiple central processing units using the Sun Grid Engine scheduling system or its equivalents such as Univa Grid Engine and multicore processing is available for subcommands composing SGAINS, in particular, for the time-consuming enumeration of uniquely mapping regions. In SCclust, most of the time is consumed by permutation tests required to evaluate pairwise dissimilarity of cell genomes, and a massive speed-up is achieved by multiple-core execution. The latter relies on functions in the R package “parallel”. Future versions of our software will include multiple central processing unit parallelization of this operation. In addition, the user can save time by using one of several genomic binning schemes provided with the pipeline and corresponding to the most up-to-date and widely used versions of the human and mouse genomes.

The pipeline software is written in the Python and R languages and incorporates third-party tools, including HISAT2,^8^ Bowtie,^9^ Samtools,^17^ Dask,^18^ and PyQt.^19^ Given this heterogeneity, we rely on Anaconda as our software management and distribution tool.

## DISCUSSION

The area of single-cell genomics is undergoing rapid technologic innovation. In designing the pipeline as described, we sought to ensure that data analysis, interpretation, and visualization do not become a rate-limiting factor in this development, nor an obstacle to the adoption of single-cell DNA technologies. We therefore provided a complete, extensively documented, and easily deployable solution for all steps in the analysis of sparse single-cell genomic data. Our tools were conceived initially to meet the needs of our in-house single-cell technology development^3,4,20^ and its applications to cancer.^1,2,21^ In particular, this work gave rise to novel methods for collective analysis of multiple single-cell CN profiles, described in Results, including representation of CN profiles in terms of CP-based features, the definition of dissimilarity between CN profiles, and the statistically supported identification of clones. These were later adapted for use by a broader research community, including users of commercial single-cell genomics platforms such as those provided by 10× Genomics. Their subsequent evolution will be shaped, in large measure, by community feedback.

In developing our visualization tool, the SCGV, we emphasized the integration of genomic data with anatomic and histopathologic annotation. The value of such integration is illustrated in Fig 3A, where cells composing the cancer clone are readily seen to originate from areas of the prostate where high-grade (Gleason score 9) cancer tissue was found on pathologic examination. Moreover, 2 such areas are each dominated by a distinct cancer subclone.

We foresee a number of important directions in which our software will evolve in the future. First, there is additional information to be extracted from single-cell genomic data and used for more accurate reconstruction of the clonal structure. In particular, if germline single-nucleotide sequence variants are known for the patient, these can be used to infer the allele-specific CN profile of each cell. Patterns of somatic single-nucleotide variation, observed in single cells, contain information about the clonal structure and can be used for its inference,^22,23^ in combination with the CN data, especially in cancer types in which such variation is common. Second, by integrating genomic and transcriptional single-cell data,^24^ both with anatomic and histopathologic annotation, we are likely to gain insights into the properties of tumors that are beyond reach of any one of these modalities alone. For example, it would be of great interest to learn the associations between tumor clonal populations and elements of the microenvironment. We will address algorithmic learning and visualization for this purpose in future work. The SGAINS pipeline, the SCclust package, the SCGV graphical interface, and the input data for the examples of SCGV use discussed in Results are available for download.^25^
